# Detecting Algorithmic Errors and Patient Harms for AI-Enabled Medical Devices in Randomized Controlled Trials: Protocol for a Systematic Review

**DOI:** 10.2196/51614

**Published:** 2024-06-28

**Authors:** Aditya U Kale, Henry David Jeffry Hogg, Russell Pearson, Ben Glocker, Su Golder, April Coombe, Justin Waring, Xiaoxuan Liu, David J Moore, Alastair K Denniston

**Affiliations:** 1 Institute of Inflammation and Ageing University of Birmingham Birmingham United Kingdom; 2 University Hospitals Birmingham NHS Foundation Trust Birmingham United Kingdom; 3 NIHR Birmingham Biomedical Research Centre Birmingham United Kingdom; 4 NIHR Incubator for AI and Digital Health Research Birmingham United Kingdom; 5 Population Health Science Institute Faculty of Medical Sciences Newcastle University Newcastle upon Tyne United Kingdom; 6 Medicines and Healthcare Products Regulatory Agency London United Kingdom; 7 Kheiron Medical Technologies London United Kingdom; 8 Department of Computing Imperial College London London United Kingdom; 9 Department of Health Sciences University of York York United Kingdom; 10 Institute of Applied Health Research University of Birmingham Birmingham United Kingdom; 11 Health Services Management Centre University of Birmingham Birmingham United Kingdom

**Keywords:** patient safety, adverse events, randomized controlled trials, medical device, systematic review, algorithmic, artificial intelligence, AI, AI health technology, safety, algorithm error

## Abstract

**Background:**

Artificial intelligence (AI) medical devices have the potential to transform existing clinical workflows and ultimately improve patient outcomes. AI medical devices have shown potential for a range of clinical tasks such as diagnostics, prognostics, and therapeutic decision-making such as drug dosing. There is, however, an urgent need to ensure that these technologies remain safe for all populations. Recent literature demonstrates the need for rigorous performance error analysis to identify issues such as algorithmic encoding of spurious correlations (eg, protected characteristics) or specific failure modes that may lead to patient harm. Guidelines for reporting on studies that evaluate AI medical devices require the mention of performance error analysis; however, there is still a lack of understanding around how performance errors should be analyzed in clinical studies, and what harms authors should aim to detect and report.

**Objective:**

This systematic review will assess the frequency and severity of AI errors and adverse events (AEs) in randomized controlled trials (RCTs) investigating AI medical devices as interventions in clinical settings. The review will also explore how performance errors are analyzed including whether the analysis includes the investigation of subgroup-level outcomes.

**Methods:**

This systematic review will identify and select RCTs assessing AI medical devices. Search strategies will be deployed in MEDLINE (Ovid), Embase (Ovid), Cochrane CENTRAL, and clinical trial registries to identify relevant papers. RCTs identified in bibliographic databases will be cross-referenced with clinical trial registries. The primary outcomes of interest are the frequency and severity of AI errors, patient harms, and reported AEs. Quality assessment of RCTs will be based on version 2 of the Cochrane risk-of-bias tool (RoB2). Data analysis will include a comparison of error rates and patient harms between study arms, and a meta-analysis of the rates of patient harm in control versus intervention arms will be conducted if appropriate.

**Results:**

The project was registered on PROSPERO in February 2023. Preliminary searches have been completed and the search strategy has been designed in consultation with an information specialist and methodologist. Title and abstract screening started in September 2023. Full-text screening is ongoing and data collection and analysis began in April 2024.

**Conclusions:**

Evaluations of AI medical devices have shown promising results; however, reporting of studies has been variable. Detection, analysis, and reporting of performance errors and patient harms is vital to robustly assess the safety of AI medical devices in RCTs. Scoping searches have illustrated that the reporting of harms is variable, often with no mention of AEs. The findings of this systematic review will identify the frequency and severity of AI performance errors and patient harms and generate insights into how errors should be analyzed to account for both overall and subgroup performance.

**Trial Registration:**

PROSPERO CRD42023387747; https://www.crd.york.ac.uk/prospero/display_record.php?RecordID=387747

**International Registered Report Identifier (IRRID):**

PRR1-10.2196/51614

## Introduction

### Background

Artificial intelligence (AI), the use of machines to undertake complex processes that would usually require human intelligence, has the potential to transform health care [1,2]. The potential benefits of such data-led technologies include a wide range of clinical applications, such as faster diagnosis, prognostics, digital therapeutics, and even the detection of novel signals [3-5]. Although there has been a great deal of enthusiasm around AI medical devices, performance in computer-based test environments is often different from that in the real world [6-8]. There is an urgent need to investigate how such technologies can be evaluated and monitored to ensure clinical benefit and avoid patient harm [9-12].

### AI Errors and Patient Harms

The translation of AI medical devices from “code to clinic” is complex and, if planned poorly, can lead to serious safety concerns [13,14]. Safety assessments involve understanding risks associated with AI medical devices, including what AI errors can arise, how these might lead to patient harms, and what failure modes may exist. These concepts are defined in Textbox 1.

**Glossary of terms.**

**Adverse events**

- “An unfavourable outcome that occurs during or after the use of a drug or other intervention but is not necessarily caused by it” [15,16]

**Artificial intelligence (AI) errors**

- “Any outputs of the AI system which are inaccurate, including those which are inconsistent with expected performance and those which can result in harm if undetected or detected too late.” [9]

**Failure modes**

- “The tendency to malfunction in the presence of certain conditions. Whereas an error can be a single occurrence, failure modes represent errors which will repeatedly occur and often have similar consequences.” [9]

**Patient harms**

- “Injury or damage to the health of people” (as defined in International Organization for Standardization [ISO] 14971- application of risk management for medical devices) [17]
- “The totality of possible adverse consequences of an intervention or therapy” [18]

### Performance Evaluation and Monitoring of AI Medical Device

AI medical device safety and effectiveness evidence can be generated at various stages in the evaluation process, which can be broadly divided into pre- and postmarket evaluations. Premarket evaluation includes a range of study types such as test accuracy studies and randomized controlled trials (RCTs). Postmarket evaluation on the other hand includes these study types in addition to local assurance practices and ongoing monitoring. Several study designs exist for the generation of effectiveness evidence, with the most robust evidence in terms of minimizing bias and objectively measuring the effect of AI interventions on clinical outcomes being derived from prospective RCTs [19]. Recent literature demonstrates the importance of in-depth performance error analysis including the identification of “inhuman errors” (eg, highly displaced fractures missed by AI), testing for algorithmic encoding of protected characteristics, and conducting exploratory error analyses to identify cases of hidden stratification [20-22]. An AI medical device might be shown to perform well overall; however, without more rigorous error analysis including exploratory and subgroup analysis, it is not possible to truly understand the clinical impact on patients as individuals. The concept of performance error analysis has been outlined in the recent AI extension reporting guidelines for clinical trials and trial protocols (Consolidated Standards of Reporting Trials-AI [CONSORT-AI] and The Standard Protocol Items: Recommendations for Interventional Trials-AI) [23,24]. Recent systematic reviews demonstrate that the quality of reporting of RCTs remains both suboptimal and variable [25,26]. The reviews demonstrated poor adherence of published RCTs to the CONSORT-AI reporting guidelines. There is still minimal literature specifically describing the reporting and analysis of errors and adverse events (AEs), and how performance error analysis is being conducted. There is a need to conduct a literature review in this area to inform future clinical evaluations of AI medical devices and real-world AE reporting. This systematic review aims to explore AI errors and AE reporting in RCTs of AI interventions.

### Purpose

This systematic review will assess the frequency and severity of AI errors and AEs in RCTs investigating AI medical devices as interventions in clinical settings. Where reported, data regarding AI system risks, reported errors, and how these errors were analyzed will be extracted. Our research question is, what are the characteristics (including frequency and severity) of AI errors and AEs in RCTs and how are these performance errors analyzed?

### Aim

The primary aims of this review are to assess the frequency, severity, and types of errors and AEs reported in RCTs of AI medical devices. The secondary aims of the review include (1) identifying what analyses are conducted when errors or harms are reported and (2) reporting the error and AE detection methods used.

## Methods

### Protocol

**Figure 1:**
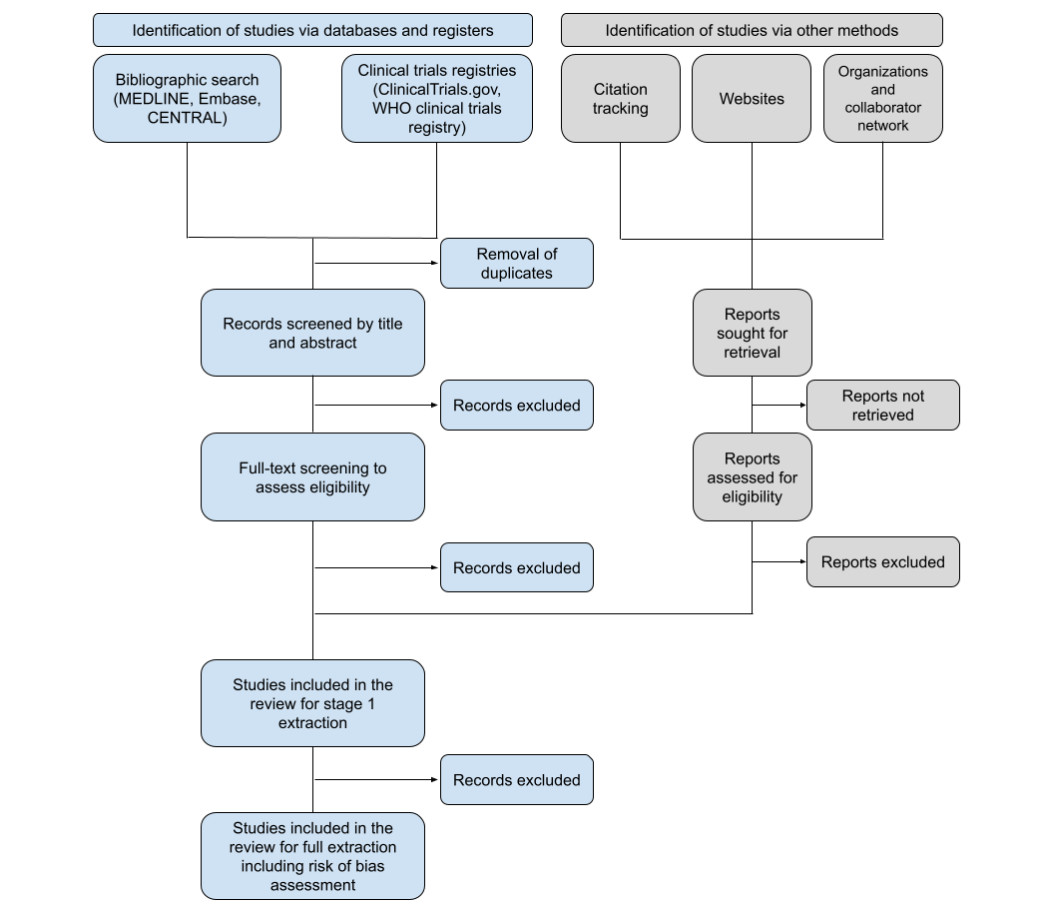
PRISMA flow diagram outline to be populated during the systematic review process. PRISMA: Preferred Reporting Items for Systematic Reviews and Meta-Analyses; WHO: World Health Organization.

This systematic review protocol is written in compliance with the PRISMA-P (Preferred Reporting Items for Systematic Review and Meta-Analysis Protocol) guidelines [27]. The completed systematic review will be reported in line with PRISMA (Preferred Reporting Items for Systematic Reviews and Meta-Analyses) guidance (Figure 1) [28]. Preferred Reporting Items for Systematic Reviews and Meta-Analyses Artificial Intelligence (PRISMA-AI) will be used if published before the submission of this systematic review [29].

### Systematic Review Registration

This systematic review protocol is registered on PROSPERO (CRD42023387747).

### Information Sources

The search strategy will be used to search 3 online bibliographic databases, in addition to clinical trial registries, to identify RCTs evaluating AI interventions in clinical settings. Literature searches will not be limited by year to ensure that all AI medical device RCTs are identified. The bibliographic databases of published studies are MEDLINE, Embase, and Cochrane CENTRAL. The registers of clinical trials are ClinicalTrials.gov and the World Health Organization (WHO) International Clinical Trials Registry Platform (ICTRP portal).

### Search Strategy

In bibliographic databases, free text, and index terms will be used to search for RCTs of AI medical devices. Clinical trial registries will be searched using in-built filters to identify RCTs with results. RCTs identified in bibliographic databases will be cross-referenced using clinical trial registries to ensure that all harms data are captured. The search strategy has been developed in consultation with an information specialist (AC) and further details are included in Multimedia Appendix 1 [26,30-33]. The searches were executed on June 30, 2023. No date cutoff was applied. The full search strategies are available in Multimedia Appendix 2. Reference lists of included reports will be checked to capture additional RCTs. Additionally, experts in the field will be contacted to identify reports that were not available from the databases listed above.

### Selection Criteria

#### Overview

The selection criteria are structured using the Studies, Data, Methods, Outcome measures (SDMO) framework for methodological systematic reviews that were deemed most appropriate and adapted for this study [34]. Studies not published in the English language will be included where the translation is available.

#### Types of Studies

Only RCTs will be included in this systematic review. Other study types including nonrandomized clinical trials, observational studies, and case studies will be excluded. The review will include trials where randomization happens at any level (such as cluster randomization and crossover RCTs).

#### Types of Data

AI medical device interventions that directly affect patient care will be included, for example, diagnostic, prognostic, or therapeutic tasks. AI medical devices will be included if their function, as described within the trial, is consistent with the function of a medical device, that is, within the range of functions attributed to medical devices as defined by the International Medical Device Regulators Forum (IMDRF) [35]. AI medical devices that are deployed for nonclinical tasks will be excluded. RCTs evaluating robotic interventions will also be excluded.

#### Types of Methods

RCTs with control arms involving a non-AI standard of care will be included. RCTs with only AI-enabled control arms will be excluded. Additionally, the review will include trials where error analysis has been conducted.

#### Outcomes

RCTs reporting AEs and patient harms (not explicitly reported as AEs) will be included in the final analysis. Studies not involving these outcomes will be examined to extract data related to the RCT design and characteristics of the AI medical device.

### Selection Process

Once papers have been identified through the search strategy, the studies will be screened for relevance by title and abstract. The Rayyan systematic review tool will be used to screen results [36]. Irrelevant studies will be removed. This process will be carried out by 2 reviewers independently and any discrepancies will be resolved by discussion or referral to an arbitrator.

Papers identified as potentially relevant will then be retrieved and the full text will be assessed for inclusion against the selection criteria described above. During full-text screening, the studies will also be assessed for the presence of patient harm data or any form of performance error analysis. Those with these data present will be marked for full extraction and risk of bias assessment, and those that do not report these data will be marked for the extraction of the RCT design and AI technology characteristics only. This will again be done by 2 reviewers independently with recourse to arbitration if required .

If included RCTs do not report errors or AEs, only data relating to the type of AI medical device and trial design will be extracted. This is signposted as stage 1 extraction in the PRISMA diagram. Further details are included in the data extraction section.

### Data Extraction

The data extraction process will be undertaken using a standardized, piloted data extraction form. Data will be entered into the data extraction form in Microsoft Excel. This will be done by 2 reviewers who will complete data extraction independently using the agreed data extraction template. Authors of papers will be contacted for further information and clarification where required. Where available, the following items will be extracted (Textbox 2).

**Data points for extraction using piloted data extraction form.**

**Study characteristics**

- Title, authors, publication year, journal, and country
- Specialty (medical discipline, eg, radiology, ophthalmology, or cardiology)
- Study context (eg, primary care or hospital care)
- Study design
- Sample size
- Study length (time period)
- Control arm comparator (overview of workflow)
- Baseline characteristic subgroups (eg, sex, age, ethnicity, and socioeconomic details)
- Primary and secondary endpoints

**Characteristics of the artificial intelligence (AI) medical device**

- Name of AI medical device
- AI developer (and manufacturer where relevant)
- AI subtype, for example, “recurrent neural network”
- AI-intended use and clinical pathway (context)
- AI autonomy level (ie, the extent to which human oversight is expected). The autonomy level will be graded from 1 to 5 based on the classification described in the literature [37]
- Input data
- AI output
- Role in clinical decision-making
- Characteristics of the end user (eg, clinician or patient)

**Outcomes and findings**

- Primary outcomes (to satisfy primary objectives of systematic review):
  Frequency of AI errors
  Frequency and severity of adverse events (AEs; as classified by relevant regulatory documents including International Organization for Standardization [ISO] 14971-application of risk management for medical devices) in all study arms [17]
  Characteristics of error, patient harm, and AEs identified
- Secondary outcomes (to satisfy secondary objectives of systematic review):
  Types of performance error analysis, for example, subgroup analysis by patient or task characteristics
  Error and AE detection methods described in the study and risk mitigations in place during the randomized controlled trial (RCT)

### Reporting of AEs and Performance Error Analysis

Characteristics of the AI medical device being evaluated will be extracted for all included RCTs. Full data extraction will only be completed for studies reporting some form of AEs (or possible patient harms not explicitly reported by authors) or details of performance error analysis (item 19 of the CONSORT-AI extension) [23]. Performance error analysis is defined as any of the following: (1) exploratory error analysis, (2) subgroup analysis, or (3) adversarial testing [9].

### Quality Assessment

Assessment of quality will be carried out for all included studies. Version 2 of the Cochrane risk-of-bias tool (RoB2) for randomized trials will be used to assess studies [38]. Assessment will be undertaken by 2 reviewers independently with arbitration by a third reviewer where required. The risk is categorized into “low,” “high,” or alternatively “some concerns.”

### Data Synthesis

#### Overview

Findings will be synthesized in both narrative and tabular formats. Included studies will be divided into 3 groups (1, 2a, and 2b as shown) for within-group (and between-group where possible) comparison, based on the AI medical device type and RCT study design

1. Studies assessing therapeutic AI medical devices (eg, drug-dosing algorithms and AI-enabled psychological therapies)
2. Studies assessing diagnostic or predictive AI medical devices
  - With ground truth (where ground truth is a reference test, for example, biopsy result or clinician opinion)
  - Without ground truth

The synthesis of data will be divided into 2 sections consistent with the aims outlined in this protocol. The first section is focuses on the primary aims of the review—the frequency, severity, and types of AI errors and patient harms. The second section is focused on the secondary aims of the review: (1) the reporting of harms data based on the CONSORT harms extension, (2) the types of performance error analysis described, and (3) identified subgroups of interest for each health area.

#### Analysis to Achieve Primary Aims

AI error and patient harm rates will be calculated for each RCT. These data will be compared between and within the identified groups. The analyses that will be considered are as follows:

- First, reported AEs with comparison between AI and control arms, such as (1) frequency and severity of AEs for each technology, with comparison between AI medical device groups listed; (2) whether the AE was directly linked to the AI medical device (as assessed by RCT authors); and (3) severity of AEs will be based on guidance from international standards (ISO 14971-application of risk management for medical devices) [17].
- Second, the frequency of errors, for example, false positives or false negatives for diagnostic AI medical devices. If the AI output is reported as likelihood distribution, then the analysis will be directed by the subsequent clinical action taken in response to the AI output. If a ground truth is present in the study, then a comparison can be made, such as (1) a comparison within and between AI medical device groups listed. The type of algorithm used by the AI medical device will also be included for comparison, and (2) if appropriate, a meta-analysis will be conducted investigating harms as a proportion of total outputs for intervention versus control arms. Appropriateness will be defined by assessing the heterogeneity of trial characteristics. Assessment of heterogeneity will include the consideration of trial design, primary outcomes, and the types of reported AEs.
- Third, the characterization of errors and harms for AI medical devices, such as (1) comparison between AI medical device error rate and erroneous clinical action. For example, if the AI medical device output incorrectly suggests the administration of a drug, is this drug actually administered? (2) Harms that are identified but not explicitly reported by authors will also be extracted where possible.

#### Analysis to Achieve Secondary Aims

##### Failure Modes

The number of studies describing subgroup and exploratory error analysis will be recorded. First, subgroup analysis of AI medical device performance for the clinical task will be documented. Subgroups of interest described in RCTs will be documented for each medical specialty. Second, exploratory error analysis will be documented with a specific focus on the types of scenarios most likely to cause errors for each clinical use case. Described failure modes will be documented for each medical specialty and clinical task. Third, the types of performance analysis conducted for each type of AI medical device and clinical discipline will be compared to identify groups with high rates of failure.

##### Error and AE Detection Methods

Error and AE detection methods will be recorded for each study. The extraction of AI medical device characteristics for all identified RCTs (including those excluded from full extraction) will demonstrate trends in AI medical devices with no AEs or implicit patient harms. This will allow for the identification of areas where AE detection methods are particularly underdeveloped or less frequently used. An example of an AE detection method is the use of questionnaires to allow patients to self-report AEs after interaction with an AI-enabled mental health chatbot.

## Results

The project was registered on PROSPERO in February 2023. Preliminary searches have been completed and the search strategy has been designed in consultation with an information specialist and methodologist (AC and DJM). Searches were conducted in June 2023. Title and abstract screening began in September 2023 and finished in February 2024. After deduplication, 11,913 papers were screened resulting in 423 eligible studies for full-text screening. The full-text screening was completed in April 2024. Data extraction commenced in April 2024. Data analysis and paper drafting will be conducted from May 2024 to July 2024.

## Discussion

The potential value of AI medical devices is well recognized, and numerous studies have been published recently relating to model development and evaluation [30,31]. Although AI medical devices show promise, there are still barriers to their deployment at scale. One of the most important related challenges is ensuring that these technologies are effective, safe, and inclusive. As an interventional study, RCTs allow the measurement of clinically relevant outcomes including patient harms that would not be possible in an in silico study. As a randomized clinical trial, the study design minimizes bias and is, therefore, considered the gold standard of clinical evidence.

This systematic review aims to assess the frequency and severity of AI errors and AEs. Data will be extracted regarding how AEs and AI errors are analyzed such as subgroup analysis and identification of failure modes. Investigating the severity and frequency of errors and AEs in addition to how these are reported in RCTs may provide insights into study design, real-world impacts, and methods for evaluating unintended effects of AI medical devices. The systematic review will not only shed light on which AI medical devices or RCT designs most commonly report AEs, but also on the methods used for AE detection. A summary of these methods will be an important part of the insights generated by this study. The main anticipated limitation of this systematic review is the heterogeneity of outcomes across the different medical disciplines and types of AI medical devices. This will be addressed by grouping RCTs based on the type of AI medical device and medical specialty where appropriate. The benefits of a broad review in this instance outweigh the limitations given the lack of consensus in the analysis and reporting of AI errors and AEs. Furthermore, recent literature reviews have demonstrated poor adherence to CONSORT-AI guidelines which indicates a reporting limitation. This means that if no AI errors or AEs are reported, this will not necessarily stipulate that none had occurred in the study. Finally, AI error may or may not lead to clinical error and there will be other instances where clinical error is introduced by human involvement in the workflow. Mapping clinical workflows and analyzing work system elements will be important; however, there might be reporting limitations. Where relevant, authors may be contacted for further information.

There is a growing unmet need for methods enabling the detection, analysis, and reporting of AI errors and AEs related to AI medical device usage. This systematic review aims to be the first of its kind focused on errors and AEs associated with AI medical devices in health care. The impact of this systematic review will be 2-fold. First, it will demonstrate current practices in error and AE detection, analysis, and reporting, forming the basis for further work around best practices for AI harms in RCTs. Second, we hope that this work will inform the real-world deployment of AI medical devices, particularly safety monitoring and risk mitigation practices, which is an area of significant interest globally. This will be achieved through the signposting of best practices for AE detection and performance error analysis identified through the review. This is part of a wider program of work looking at postmarket safety monitoring of AI medical devices. A complementary systematic review focusing on AEs reported in regulatory databases is also being conducted.

## Appendix

Multimedia Appendix 1 Development of search strategy for MEDLINE and Embase.

Multimedia Appendix 2 Search strategies.

## Abbreviations

AE: adverse event
AI: artificial intelligence
CONSORT-AI: Consolidated Standards of Reporting Trials-Artificial Intelligence
ICTRP: International Clinical Trials Registry Platform
IMDRF: International Medical Device Regulators Forum
ISO: International Organization for Standardization
PRISMA: Preferred Reporting Items for Systematic Reviews and Meta-Analyses
PRISMA-AI: Preferred Reporting Items for Systematic Reviews and Meta-Analyses Artificial Intelligence
PRISMA-P: Preferred Reporting Items for Systematic Review and Meta-Analysis Protocol
RCT: randomized controlled trial
SDMO: Studies, Data, Methods, Outcome measures
WHO: World Health Organization
