# Supplementary material for: Detecting Algorithmic Errors and Patient Harms for AI-Enabled Medical Devices in Randomized Controlled Trials: Protocol for a Systematic Review
Source: JMIR Res Protoc. 2024 Jun 28;13:e51614. doi: 10.2196/51614 (PMC11245650; doi:10.2196/51614)
Supplement: Multimedia Appendix 1 [file resprot_v13i1e51614_app1.docx]

**Appendix 1: Development of search strategy for MEDLINE and EMBASE**

**MEDLINE and EMBASE search terms**

The search strategy aims to identify all RCTs evaluating AI medical devices as interventions in healthcare. The initial search terms were generated in stages. First, a mind mapping exercise was undertaken to identify key search terms. Recently published systematic reviews to identify similar studies were then consulted to identify variations of terms and new search terms. These studies included: (Liu et al. 2019; Nagendran et al. 2020; Zhou et al. 2021; Lam et al. 2022; Plana et al. 2022) [26, 30-33]. Search terms were included if they were relevant to 1) artificial intelligence, 2) machine learning or 3) deep learning. The search strategy was refined using the term finder tool in OVID medline.

Second, we assessed the search terms against a corpus of RCTs that we had identified. We used a sample of 40 identified RCTs to refine the search further. This stage included removal of search terms that were generating high numbers of irrelevant search results. Examples included “fuzzy logic”, “support vector machines”, “elastic net”, “multilayer perceptron”. We then combined these terms with the Cochrane RCT filter for MEDLINE. We used the sensitivity and precision maximising filter. This is shown in Table 1 below.

After finalising the search terms, we conducted the search and ensured that all 40 RCTs were captured by the search. We then identified further terms that yielded irrelevant results. The Cochrane RCT filter, which is structured to identify RCTs evaluating drug interventions, was modified. As shown in table 1, the modifications included removal of “randomly.ab” “trial.ti” and “placebo.ab”. The rationale for this is also listed in table 1. “Randomized.ab” was adjusted to “randomi#ed.ti,ab” to take into account alternative spelling and search titles also.

Finally, we ran the search in MEDLINE and EMBASE to ensure that all 40 RCTs were captured. The search strategy was translated from MEDLINE into EMBASE using Emtree. Conference abstracts are excluded from the EMBASE search. We compared the searches between the original Cochrane RCT filter, and the modified search filter. The original filter combined with our AI/ML/DL search terms yielded 9282 hits in MEDLINE, versus 4818 with the modified filter. MEDLINE and EMBASE results for the modified filter identified all 40 RCTs.

The final stage of testing our strategy involved assessing a random sample of 100 studies that the original Cochrane RCT filter had picked up, but the modified filter had not. These studies were screened by title and abstract. Of these 100, none were RCTs evaluating clinical AI interventions.

**Cochrane CENTRAL search terms**

Given that we had identified three terms in the original Cochrane search that were yielding a significant number of irrelevant hits, we trialled application of our new search filter in the CENTRAL database. Without any RCT filters, there are 8468 RCTs identified with our search terms. With the full original Cochrane search filter, we identified 7026 studies. With our modified Cochrane search filter, we identified 1916 studies in the CENTRAL database.

To test our search strategy in CENTRAL, we took a random sample of 100 studies that were identified in the 7026 studies, and NOT in the 1916 studies. These studies were screened by title and abstract. Of these 100 studies, none were RCTs evaluating clinical AI interventions. After consultation with an information specialist and cochrane guidance, we are not using an RCT filter to search the CENTRAL database. Information regarding the search filter is included for reference**.** Full search strategies are available in appendix 2.

| 1  2  3  4  5  6  7  8  9  10 | randomized controlled trial.pt.  controlled clinical trial.pt.  randomized.ab. (this was adjusted to randomi#ed.ti,ab)  placebo.ab. (REMOVED)  clinical trials as topic.sh.  randomly.ab. (REMOVED)  trial.ti. (REMOVED)  1 or 2 or 3 or 4 or 5 or 6 or 7  exp animals/ not humans.sh.  8 not 9 | *Placebo* removed as this is specific to drug trials.  *Randomly* generated many irrelevant hits. One of the most important issues with this search term is that with data driven technologies, data is “randomly” split for training and test purposes. Nearly every paper describing AI development will have this term in the abstract.    *Trial* identified a significant number of studies that were not RCTs. Often the studies were “observational diagnostic trials” reported against STARD guidelines. |
| --- | --- | --- |

Table 1: Cochrane Highly Sensitive Search Strategy for identifying randomized trials in MEDLINE: sensitivity- and precision-maximizing version (2008 revision); Ovid format
