# Supplementary material for: Detecting Algorithmic Errors and Patient Harms for AI-Enabled Medical Devices in Randomized Controlled Trials: Protocol for a Systematic Review
Source: JMIR Res Protoc. 2024 Jun 28;13:e51614. doi: 10.2196/51614 (PMC11245650; doi:10.2196/51614)
Supplement: Multimedia Appendix 2 [file resprot_v13i1e51614_app2.docx]

The following search strategies were developed in consultation with an information specialist. Search strategies for MEDLINE, EMBASE and Cochrane CENTRAL are described below. This is the final search strategy after further refinement following on from testing (appendix 1). No further changes were made to the adapted RCT filter, however further refinement of AI/ML/DL terms was undertaken.

**CENTRAL**

| **#** | **Searches** |
| --- | --- |
| **1** | MeSH descriptor: [Artificial Intelligence] explode all trees |
| **2** | Artificial near/2 intelligence:ti,ab,kw |
| **3** | (machine near/3 learn*):ti,ab,kw |
| **4** | (deep near/3 learn*):ti,ab,kw |
| **5** | (convolutional NEXT net*):ti,ab,kw |
| **6** | (neural NEXT net*):ti,ab,kw |
| **7** | (deep NEXT net*):ti,ab,kw |
| **8** | naive bayes:ti,ab |
| **9** | random forest:ti,ab |
| **10** | multilayer perceptron:ti,ab |
| **11** | (reinforcement near/2 learn*):ti,ab |
| **12** | (semi-supervised near/2 learn*):ti,ab |
| **13** | (self-supervised near/2 learn*):ti,ab |
| **14** | CADe:ti,ab |
| **15** | computer-aided detection:ti,ab |
| **16** | predictive algorithm:ti,ab |
| **17** | AI:ti |
| **18** | #1 or #2 or #3 or #4 or #5 or #6 or #7 or #8 or #9 or #10 or #11 or #12 or #13 or #14 or #15 or #16 or #17 |

**MEDLINE**

| **#** | **Searches** |
| --- | --- |
| **1** | exp Artificial Intelligence/ |
| **2** | (Artificial adj1 intelligence).ti,ab,kw. |
| **3** | (machine adj3 learn*).ti,ab,kw. |
| **4** | (deep adj3 learn*).ti,ab,kw. |
| **5** | (convolutional adj1 net*).ti,ab. |
| **6** | (neural adj1 net*).ti,ab. |
| **7** | (deep adj1 net*).ti,ab. |
| **8** | naive bayes.ti,ab. |
| **9** | random forest.ti,ab. |
| **10** | multilayer perceptron.ti,ab. |
| **11** | (reinforcement adj2 learn*).ti,ab. |
| **12** | (semi-supervised adj2 learn*).ti,ab. |
| **13** | (self-supervised adj2 learn*).ti,ab. |
| **14** | CADe.ti,ab. |
| **15** | computer-aided detection.ti,ab. |
| **16** | predictive algorithm*.ti,ab. |
| **17** | (AI not ("aromatase inhibitor*" or "anatomic insertion*" or AI-IgG or "apnea index" or "anti infective" or "anti infective" or "angiogenesis inhibitors" or angiogenesis-inhibitors)).ti |
| **18** | 1 or 2 or 3 or 4 or 5 or 6 or 7 or 8 or 9 or 10 or 11 or 12 or 13 or 14 or 15 or 16 or 17 |
| **19** | randomized controlled trial.pt. |
| **20** | controlled clinical trial.pt. |
| **21** | randomi#ed.ti,ab. |
| **22** | clinical trials as topic.sh. |
| **23** | 19 or 20 or 21 or 22 |
| **24** | exp animals/ not humans.sh. |
| **25** | 23 not 24 |
| **26** | 18 and 25 |

**EMBASE**

| **#** | **Searches** |
| --- | --- |
| **1** | exp artificial intelligence/ |
| **2** | (artificial adj1 intelligence).tw. |
| **3** | (machine adj3 learn*).tw. |
| **4** | (deep adj3 learn*).tw. |
| **5** | (convolutional adj1 net*).tw. |
| **6** | (neural adj1 net*).tw. |
| **7** | (deep adj1 net*).tw. |
| **8** | naive bayes.tw. |
| **9** | random forest.tw. |
| **10** | multilayer perceptron.tw. |
| **11** | (reinforcement adj2 learn*).tw. |
| **12** | (semi-supervised adj2 learn*).tw. |
| **13** | (self-supervised adj2 learn*).tw. |
| **14** | CADe.tw. |
| **15** | computer-aided detection.tw. |
| **16** | predictive algorithm.tw. |
| **17** | (AI not ("aromatase inhibitor*" or "anatomic insertion" or AI-IgG or "apnea index" or "anti infective" or "angiogenesis inhibitors" or "angiogenesis-inhibitors")).ti |
| **18** | controlled clinical trial/ or "controlled clinical trial (topic)"/ |
| **19** | randomi?ed.tw. |
| **20** | randomized controlled trial/ |
| **21** | exp animal/ not human.sh. |
| **22** | 1 or 2 or 3 or 4 or 5 or 6 or 7 or 8 or 9 or 10 or 11 or 12 or 13 or 14 or 15 or 16 or 17 |
| **23** | 18 or 19 or 20 |
| **24** | 23 not 21 |
| **25** | 22 and 24 |
| **26** | limit 25 to conference abstract status |
| **27** | 25 not 26 |
